# Different Bacterial Communities Involved in Peptide Decomposition between Normoxic and Hypoxic Coastal Waters

**DOI:** 10.3389/fmicb.2017.00353

**Published:** 2017-03-07

**Authors:** Shuting Liu, Boris Wawrik, Zhanfei Liu

**Affiliations:** ^1^Marine Science Institute, The University of Texas at Austin, Port AransasTX, USA; ^2^Department of Microbiology and Plant Biology, The University of Oklahoma, NormanOK, USA

**Keywords:** peptide, bacteria, DNA stable isotope probing, nitrogen, hypoxia, Gulf of Mexico

## Abstract

Proteins and peptides are key components of the labile dissolved organic matter pool in marine environments. Knowing which types of bacteria metabolize peptides can inform the factors that govern peptide decomposition and further carbon and nitrogen remineralization in marine environments. A ^13^C-labeled tetrapeptide, alanine-valine-phenylalanine-alanine (AVFA), was added to both surface (normoxic) and bottom (hypoxic) seawater from a coastal station in the northern Gulf of Mexico for a 2-day incubation experiment, and bacteria that incorporated the peptide were identified using DNA stable isotope probing (SIP). The decomposition rate of AVFA in the bottom hypoxic seawater (0.018–0.035 μM h^-1^) was twice as fast as that in the surface normoxic seawater (0.011–0.017 μM h^-1^). SIP experiments indicated that incorporation of ^13^C was highest among the Flavobacteria, Sphingobacteria, Alphaproteobacteria, Acidimicrobiia, Verrucomicrobiae, Cyanobacteria, and Actinobacteria in surface waters. In contrast, highest ^13^C-enrichment was mainly observed in several Alphaproteobacteria (*Thalassococcus, Rhodobacteraceae, Ruegeria*) and Gammaproteobacteria genera (*Colwellia, Balneatrix, Thalassomonas*) in the bottom water. These data suggest that a more diverse group of both oligotrophic and copiotrophic bacteria may be involved in metabolizing labile organic matter such as peptides in normoxic coastal waters, and several copiotrophic genera belonging to Alphaproteobacteria and Gammaproteobacteria and known to be widely distributed may contribute to faster peptide decomposition in the hypoxic waters.

## Introduction

Proteins and peptides are key components of labile dissolved organic matter (DOM) that supports bacterial growth (Azam, 1998). Small peptides (ca. <600 Da) are key immediate products of microbial protein decomposition owing to the size constraints of bacterial cell membrane transport systems, i.e., porins (Weiss et al., 1991). After proteins are degraded to small peptides, these small peptides can be either taken up directly by bacteria, or hydrolyzed further to individual amino acids via extracellular enzymes with subsequent uptake. The interaction between peptide decomposition and bacteria plays an important role in the cycling of carbon and nitrogen, regeneration of nutrients, and preservation of refractory dissolved organic nitrogen (DON) in marine environments (Aluwihare et al., 2005; Nagata, 2008).

Our previous studies have demonstrated that small peptides decompose more quickly in bottom hypoxic than surface normoxic (normal oxygen-saturated) waters in the northern Gulf of Mexico (nGOM), and that the growth of certain bacterial genera such as *Vibrio, Marinobacterium, Neptuniibacter, Pseudoalteromonas, Thalassomonas, Amphritea, Roseobacter* and *Ruegeria*, appears to respond to peptide addition (Liu et al., 2013; Liu and Liu, 2016). These results suggest that some bacterial groups may be more effective at metabolizing peptide-derived organic matter in hypoxic seawater, but direct evidence linking specific bacterial lineages to peptide decomposition has not been reported. Knowing which types of bacteria metabolize peptides may be useful in the assessment of factors that control hypoxia formation, as decomposition of labile organic matter leads to consumption of dissolved oxygen (DO) (Wright et al., 2012; Liu et al., 2013). As succession of microbial communities often occurs along with development of oxygen minimum zones (Crump et al., 2007; Zaikova et al., 2010; Parsons et al., 2015), studying the response of microbial communities to labile organic matter at different DO levels can also provide clues about linkages between microbial niche specialization and their resource utilization (Nelson and Wear, 2014).

Previous studies have demonstrated that some bacterial groups can outcompete others during the utilization of labile DOM (Eilers et al., 2000; Teske et al., 2011; Liu et al., 2015). For instance, the bacterial community shifted to Alphaproteobacteria and Betaproteobacteria dominated phylotypes in mesocosm tanks with diatom blooms that produced labile proteins, peptides and polysaccharides exudates (Murray et al., 2007). After bovine serum albumin (BSA) amendment, Gammaproteobacteria became the dominant bacterial class in the Chesapeake Bay water, while Bacteroidetes became dominant in the lower Delaware Bay water (Harvey et al., 2006). However, phylogeny-based incubation studies provide only indirect evidence of the role that different bacterial groups play in labile DOM mineralization, and only a few studies to date have linked specific bacteria groups with labile DOM decomposition directly using radioisotope-labeled substrate and microautoradiography combined with fluorescent *in situ* hybridization (MAR-FISH) technique (Tabor and Neihof, 1982; Ouverney and Fuhrman, 1999). For example, Cottrell and Kirchman (2000) identified that Bacteroidetes and Gammaproteobacteria actively utilized ^3^H-labeled protein in two estuarine waters. While powerful, hybridization techniques such as MAR-FISH are a targeted approach and require the design of unique probes to detect individual phylogenetic groups. These techniques often do not allow the identification of active bacteria beyond limited taxonomic depth due to probe hybridization constraints. In contrast, DNA-stable isotope probing (SIP) techniques provide an opportunity to interrogate activity *in situ* and to identify bacteria at several phylogenetic levels without *a priori* selection of specific phylotypes. SIP techniques were initially applied to identify bacteria that can degrade one-carbon (C_1_) compounds or specific pollutants in many environmental studies, such as discovering novel bacteria that degrade methanol, toluene, or alkanes in soils, sediments or marine seeps (Radajewski et al., 2000; Neufeld et al., 2007b; Luo et al., 2009; Redmond et al., 2010; Kleindienst et al., 2014). More recently, the application of DNA-SIP has been extended to marine environments. Examples include studies of urea uptake by marine pelagic bacteria and archaea in Arctic water, comparing bacteria incorporating glucose and cyanobacteria exudates in the Sargasso Sea, and exploring acetate-utilizing bacteria at the oxic–anoxic interface in the Baltic Sea (Gihring et al., 2009; Wawrik et al., 2009; Nelson and Carlson, 2012; Wawrik et al., 2012a; Berg et al., 2013; Connelly et al., 2014), demonstrating the utility of SIP to understand the marine C and N cycles.

The objective of this study was to gain insight into the identities of bacteria that utilize peptides in seawater. In particular, nGOM normoxic and hypoxic waters were targeted because they are characterized by contrasting biogeochemical processes and are known to harbor different bacterial communities that differentially respond to peptide addition. The^13^C-labeled tetrapeptide alanine-valine-phenylalanine-alanine (AVFA) was used as a model compound and incubated in both surface normoxic and bottom hypoxic seawater in the nGOM. The AVFA sequence is derived from the protein sequence of the large subunit of ribulose-1,5-biphosphate carboxylase/oxygenase (RuBisCO) that is ubiquitous in photosynthesis and has been used to investigate peptide hydrolysis (Liu et al., 2010, 2015; Orellana and Hansell, 2012; Liu and Liu, 2014, 2015). Although individual peptides are often undetectable in natural seawater due to their rapid turnover, they play an important role in supporting bacterial growth as intermediates released from sloppy-feeding or lysis of cells (Nagata, 2008; Sipler and Bronk, 2015).

## Materials and Methods

### Seawater Sampling

Surface (2 m) and bottom (16 m) seawater were collected at Sta. C6 (28°52′ N, 90°30′ W) in the nGOM during a May 2013 cruise on the R/V *Pelican*. This station, with a depth of 18 m and ca. 20 km offshore, is heavily influenced by Mississippi River discharge and often subjected to hypoxia during summer (Rabalais et al., 2001). Seawater was sampled using 10 L Niskin bottles mounted on a conductivity-temperature-depth (CTD) rosette (Seabird 911). Temperature, salinity, DO and chlorophyll *a* of seawater were monitored through the CTD device (**Table 1**). Seawater was filtered immediately onboard through a 0.2 μm Nylon filter (diameter 47 mm, Whatman) and preserved under -20°C for the analysis of dissolved organic carbon (DOC), total dissolved nitrogen (TDN), total dissolved amino acids (TDAAs), dissolved combined amino acids (DCAAs), dissolved free amino acids (DFAAs) and nutrients.

**Table 1:** Chemical parameters of initial surface (2 m) and bottom (16 m) seawater at Sta. C6.

Depth	Temp (°C)	Salinity (ppt)	DO (mg⋅L^-1^)	Chl *a* (μg⋅L^-1^)	DOC (μM)	TDN (μM)	DCAA (μM)	DFAA (μM)	NO_3_^-^ (μM)	NO_2_^-^ (μM)	P_i_ (μM)
2 m	25.5	27	7.9	1.51	233.3	14.3	1.79	0.18	0.54	ud	0.11
16 m	22.3	35	0.4	0.63	200.0	10.7	0.56	0.07	6.85	0.54	0.89

*ud, under detection limit (ca. 0.03 μM).*

### Peptide Incubation

Peptides ^12^C-AVFA and ^13^C-AVFA were custom-synthesized (C.S Bio, Menlo Park, CA, USA), and had a >95% compound purity (Liu et al., 2013). In ^13^C-AVFA, 17 (all three carbons in A, all five carbons in V and six carbons of the aromatic ring in F) out of total 20 carbon atoms were labeled isotopically. As DNA of specific bacteria groups might only be partially labeled (<100%) with ^13^C, bacteria groups incorporating ^13^C can be spread through all SIP fractions with different density (see related method below). The unlabeled ^12^C-AVFA incubation was thus included as a reference for comparison with ^13^C-AVFA incubation to confirm isotopic enrichment in DNA. The unlabeled SIP gradient should contain less DNA in the more dense fractions than that in the samples incubated with ^13^C substrates (Neufeld et al., 2007a). AVFA was incubated onboard in the surface normoxic and bottom hypoxic seawater. Briefly, either ^12^C-AVFA or ^13^C-AVFA was respectively amended in a series of 125 mL amber round bottles filled with 120 mL seawater at final concentrations of 0.25–0.47 μM. Duplicate incubations were conducted in the dark for 48 h at 24°C, close to the ambient seawater temperature (**Table 1**). At different time points (0, 8, 13, 24, and 48 h), 1 mL aliquots of unfiltered water were collected and fixed with formaldehyde at a final concentration of 3% and stored at 4°C for bacterial abundance analysis. The remaining 119 mL were filtered through the 0.2 μm Nylon filter and preserved at -20°C for the analysis of peptides, amino acids, ammonium, and orthophosphate (P_i_). The filters were preserved in 750 μL 1× STE (10 mM Tris-HCl [pH 8.0], 0.1 M NaCl, 1 mM EDTA [pH 8.0]) buffer at -20°C for DNA extraction and sequencing. DO was not monitored throughout the incubation, but the parallel incubation experiment showed that it remained relatively constant throughout the 72 h (Liu and Liu, 2016). Two kinds of controls were included for the incubation experiment: a seawater control without peptide amendment and a killed control with 0.48–0.58 μM ^12^C-AVFA and 180 μM HgCl_2_ to inhibit bacterial activity (Lee et al., 1992). The incubation and aliquot sampling for the controls followed the same procedures as described above, but only AVFA was analyzed in the killed control.

### Chemical Analyses

DOC and TDN of the filtered initial seawater were analyzed using a Shimadzu total organic carbon (TOC-V) analyzer coupled with a TNM-1 TDN analyzer with <6% error between duplicates (**Table 1**). DFAA were calculated as sum of individual amino acids analyzed in high performance liquid chromatography (HPLC, Shimadzu Prominence) equipped with a fluorescence detector after pre-column *o*-phthaldialdehyde (OPA) derivatization (Lindroth and Mopper, 1979; Lee et al., 2000). TDAA were analyzed in the same way as DFAA but after hydrolysis in 6 N HCl under nitrogen at 110°C for 20 h (Kuznetsova and Lee, 2002). DCAA were calculated as TDAA subtracting DFAA. Nitrate, nitrite and orthophosphate (P_i_) were measured following established protocols (Strickland and Parsons, 1968; Jones, 1984).

Alanine-valine-phenylalanine-alanine was analyzed in an HPLC-mass spectrometry (HPLC-MS) system (Shimadzu Prominence) following the method in Liu and Liu (2014). In brief, the mobile phase A was 10 mM ammonium acetate and mobile phase B was methanol. Samples were eluted through a C_18_ column (Alltima 5 μm, 150 mm × 4.6 mm) and a six-way valve was programmed to direct the sea salt peak to waste before introducing the AVFA peak to the MS detector that is equipped with an electrospray ionization (ESI) source and a quadrupole mass analyzer. ^12^C-AVFA and ^13^C-AVFA were quantified in positive ion mode under selective ion monitoring (SIM) at m/z = 407 and 424, respectively.

Alanine-valine-phenylalanine-alanine hydrolysis products including peptide fragments (AV, VF, FA, VFA) and amino acids (A, V, F) were analyzed by HPLC after pre-column OPA derivatization (Liu et al., 2013). Standard deviations of amino acid analysis among replicates were 10–20%. Ammonium, a main metabolite of AVFA, was analyzed using HPLC with post-column OPA derivatization (Gardner and St. John, 1991).

### Bacterial Abundance Analysis

Bacterial cells in the formaldehyde-preserved samples were stained with SYBR Green II (Molecular Probes, 1:100 v/v) and enumerated in a flow cytometer (BD Accuri C6) under blue laser excitation at 488 nm (Marie et al., 1997; Liu et al., 2013). Bacterial cells were counted in a fixed volume mode with a flow rate below 300 events per second and cell counts were determined in a dot plot of side scatter (SSC-H) vs. green fluorescence signal (FL1-H) on a logarithmic scale.

### DNA Extraction and Ultracentrifugation in CsCl Gradients

DNA was extracted from filtered cells using MoBio PowerSoil^®^ DNA isolation kits (MoBio Laboratories, Carlsbad, CA, USA). Leftover STE buffer was spun in a centrifuge and the supernatant was discarded. The pellet was re-dissolved in 100 μL lysis solution from MoBio Powerbead tube and then combined with filter in the tube for DNA extraction. A subsample (ca. 10 μL) at each incubation time point was saved for microbial community structure analysis (unamended control 0 h, one duplicate of ^12^C-AVFA 2 m 0 h, and one duplicate of ^12^C-AVFA 16 m 0 h samples were lost during DNA extraction), and the remainder (ca. 80 μL) was for the ultracentrifugation in CsCl gradients. Duplicate DNA samples (each account for ca. 89% (80 μL out of 90 μL) of extracted DNA) from all three time points (13, 24, and 48 h) for surface and bottom seawater incubations respectively were pooled (i.e., six samples were pooled) to obtain sufficient DNA for ultracentrifugation and fractionation. The pooled DNA was precipitated using isopropanol, and the DNA pellet was then re-suspended in 50 μL TE buffer (50 mM Tris-HCl, 15 mM EDTA [pH 8.0]) as previously described (Wawrik et al., 2009). Four pooled DNA samples (^12^C-AVFA surface, ^13^C-AVFA surface, ^12^C-AVFA bottom, ^13^C-AVFA bottom) were prepared for ultracentrifugation. Note that ultracentrifugation and following fractionation were not performed on killed control samples and unamended samples. CsCl gradient ultracentrifugation and fractionation followed protocols as described previously (Buckley et al., 2007; Luo et al., 2009; Wawrik et al., 2012a). In brief, 61–170 ng DNA, quantified through a Qubit^®^ 2.0 Fluorometer (Life Technologies), were mixed with 0.26 mL TE buffer and 4.45 mL of 1.295 g mL^-1^ CsCl in gradient buffer A (15 mM Tris-HCl [pH 8.0], 15 mM KCl, 15 mM EDTA [pH 8.0], 2 mg mL^-1^ ethidium bromide) in 4.7 mL polyallomer Optiseal tubes (Beckman). The tubes were centrifuged in a Beckman rotor VTi 65.2 at ca. 140,000 × *g* for 48 h. After ultracentrifugation, 30 150 μL fractions were collected from each tube in a Beckman fraction recovery system by replacing samples with mineral oil on top of the tubes at a constant rate using a peristaltic pump. The density of each fraction was calculated based on the refractive index that was measured in a Reichert AR200 refractometer (Wawrik et al., 2009). DNA was purified from each CsCl fraction by isopropanol precipitation and dissolved in 50 μL sterile nuclease-free water.

### Quantitative PCR (qPCR) of 16S rRNA Gene

The purified DNA from each SIP fraction was used to determine 16S rRNA gene copy numbers of bacteria via quantitative polymerase chain reaction (qPCR). Primers were 27F (5′-AGA GTT TGA TCM TGG CTC AG-3′) and 519R (5′-GWA TTA CCG CGG CKG CTG-3′) (Nakatsu and Marsh, 2007). Every 30 μL reaction mix for qPCR included 13.9 μL 2X Power SYBR Green PCR master mix (Applied Biosystems), 13.9 μL nuclease-free water, 200 nM (final concentration) of each primer, and 2 μL DNA template. qPCR was conducted in a real-time PCR system (Applied Biosystems, ABI 7300) followed the program: 2 min at 50°C, 8 min at 95°C, 40 cycles of 30 s at 95°C, 1 min at 55°C and 1 min at 72°C. Genomic DNA of *Roseobacter denitrificans* Och 114 (DSMZ 7001) was used as the standard DNA for bacteria (standard concentrations ranging from 10^-4^ to 10 ng μL^-1^). The qPCR detection limit for bacteria was ca. 0.002 ng (corresponding gene copy number of 442) and cycle threshold was ca. 31. The qPCR data was then normalized to the highest quantities of 16S rRNA gene copy numbers observed among all fractions in that gradient, in order to account for the differential abundances and distributions of their DNA in gradients (Wawrik et al., 2009; Connelly et al., 2014). These normalized numbers were named as ratios of quantities in which the highest normalized frequency measured equaled 1.

### 16S rRNA Gene PCR, Barcoding, and Illumina Sequencing

DNA from each incubation time point and each fraction collected from SIP gradients was amplified by PCR using Phusion high-fidelity DNA polymerase (Thermo Scientific) and barcoded for Illumina sequencing. PCR reactions utilized the universal forward primer 519F containing a 5′ M13 tag (5′-GTA AAA CGA CGG CCA GCA CMG CCG C-3′) and the reverse primer Bac-785R (5′-TAC NVG GGT ATC TAA TCC-3′) as previously described (Wawrik et al., 2012b; Klindworth et al., 2013). PCR started with 94°C for 2 min, followed by 28–32 cycles of 95°C for 30 s, 52.8°C for 30 s, and 72°C for 30 s, then 72°C for 5 min. The number of PCR cycles was optimized based on qPCR results and agarose gel check of PCR products to make sure enough PCR products were obtained but not reaching PCR plateau. PCR products were purified by QIAquick PCR purification kit (Qiagen) and barcoded using a unique M13-contianing primer for each sample that contained a 12 bp barcode for bioinformatical parsing of data (Wawrik et al., 2012b). Barcode tagging was checked by gel electrophoresis, amplifications were mixed at equimolar ratios, and sent to the Oklahoma Medical Research Foundation for MiSeq PE250 Illumina sequencing. Sequence reads were trimmed to a quality score of Q30 and adapter and primer sequences were trimmed from raw Illumina sequences. Overlapping forward and reverse reads were stitched and all non-overlapping sequence reads were discarded. Processed sequences were clustered into OTUs using UCLUST, checked for chimeras using USEARCH and classified into taxonomy through the QIIME pipeline (Caporaso et al., 2010b). A randomly chosen set of representative sequences from each OTU was aligned to the SILVA small-subunit rRNA reference alignment^1^ using the PyNAST algorithm (Caporaso et al., 2010a). Sequences were assigned to the genus level at the 95% identity as a compromise between resolution and conservative interpretation due to the short reads (250 bp) used here (Connelly et al., 2014). Sequences were deposited in National Center for Biotechnology Information (NCBI) GenBank under BioProject accession number PRJNA297372.

Bacterial community structures (relative abundance of genera presented in percentage) of the initial samples were assumed to be the same among unamended control, ^12^C-AVFA and ^13^C-AVFA samples. As unamended control and one duplicate of the ^12^C-AVFA 0 h samples were lost, initial bacterial community data of ^13^C-AVFA samples was more reliable to represent the initial bacterial community structure of all. To be consistent with the pooled SIP samples, results of the bacterial community structures from the three time points (13, 24, and 48 h) were also pooled and compared with others by non-metric multidimensional scaling (NMDS) using Matlab^®^. Analysis of similarity (ANOSIM) was applied to compare the bacterial community structures between surface and bottom seawater and between unamended control and AVFA treatment time-point samples using vegan package in R (Oksanen et al., 2016).

Calculating percentage enrichment of each bacterial taxa in the ^13^C-AVFA samples relative to the ^12^C-AVFA SIP samples followed the protocol of Bell et al. (2011). In brief, 16S rRNA gene copy numbers for each SIP fraction were quantified through qPCR. Then the proportion of each bacterial taxonomic group sequences in a given density range was multiplied by the 16S rRNA gene copy number in that same density range, and the derived copy number of each bacterial taxonomic group was normalized to total gene copy number within that density range to correct for the slight difference in total DNA between the ^12^C-AVFA and ^13^C-AVFA samples and this normalized gene copy number was referred as relative gene copy number. Percentage enrichment of a certain bacterial taxonomic group was defined as dividing the difference of the relative copy number summed in the heavy density fractions between the ^13^C-AVFA samples and the ^12^C-AVFA samples by the relative copy number in the ^12^C-AVFA samples within the same density range. The percentage enrichment was used as an indicator of the relative amount of ^13^C enrichment among bacterial groups. A higher percentage enrichment in one bacterial taxa relative to other taxa indicates increased cell replication responding to amended ^13^C-AVFA. For example, 100% enrichment of a given taxonomic group in the same density range indicates that its 16S rRNA gene copy number in the ^13^C treatment is twice as abundant as that in the ^12^C treatment. To estimate the error/noise level of the percentage enrichment, 95% confidence interval was calculated from all positive percentage enrichment values among bacterial taxa for both surface and bottom samples.

## Results

### Peptide Decomposition

**FIGURE 1:**
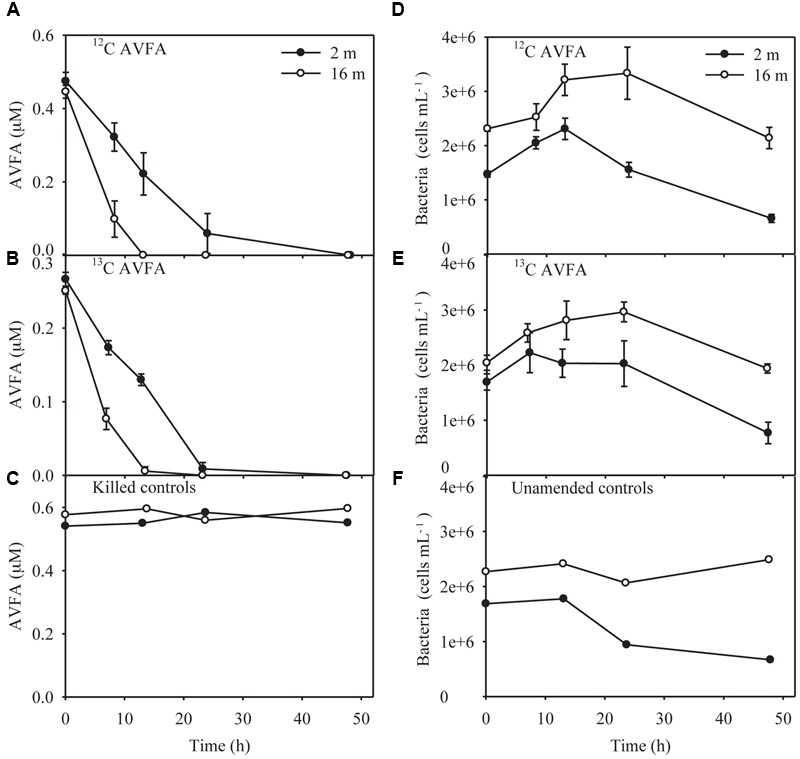
**Alanine-valine-phenylalanine-alanine (AVFA) concentrations (A–C)** and bacterial abundance counted in flow cytometer **(D–F)** with incubation time in the surface 2 m and bottom 16 m seawater of ^12^C-AVFA, ^13^C-AVFA, and control samples. Note controls samples in **(C)** are killed controls, and in **(F)** are unamended (no-AVFA) controls. Data points were presented as average ± absolute error of duplicate samples except control samples.

The ^12^C- and ^13^C-AVFA decomposition patterns were nearly identical during the 48 h incubation, as expected (**Figures 1A,B**). The AVFA concentrations decreased linearly with time in both the surface (2 m) and bottom (16 m) seawater, but the decomposition rate in the bottom seawater (0.018–0.035 μM h^-1^) was twice as high as in the surface seawater (0.011–0.017 μM h^-1^). The decomposition rate of AVFA in the bottom seawater was significantly higher than that in the surface seawater (*t*-test, *p* < 0.03). AVFA was completely degraded within 24–48 h in the surface water and within 13–24 h in the bottom water. In contrast, AVFA concentration in the killed control remained nearly unchanged during the 48 h incubation (**Figure 1C**), indicating that the peptide disappearance in the seawater was due to microbial activity.

**FIGURE 2:**
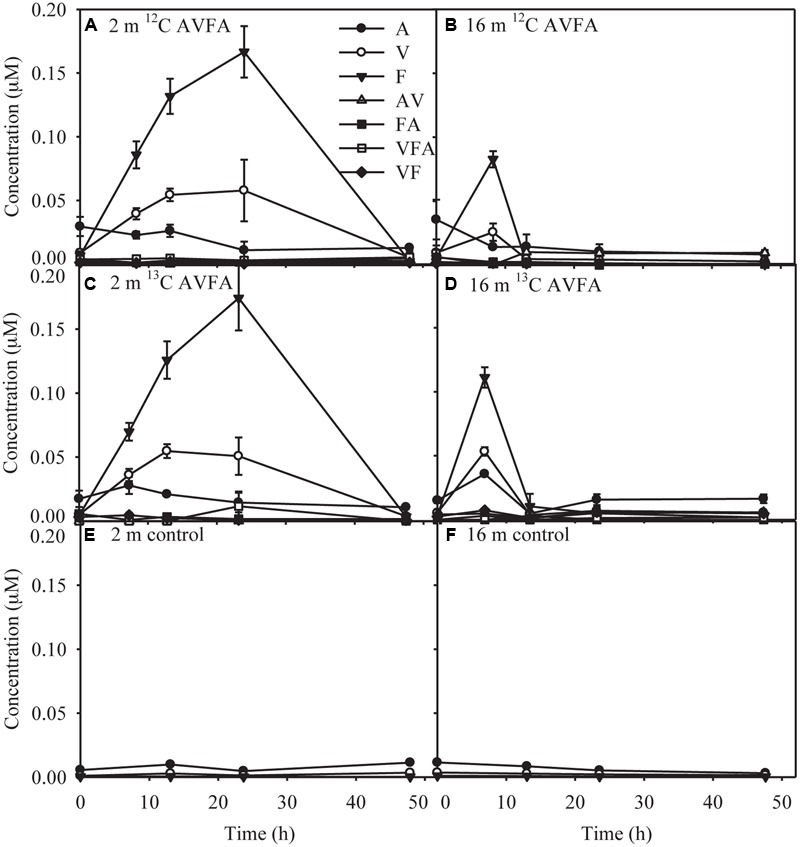
**Concentrations of produced amino acids and peptide fragments with incubation time in the (A)** surface 2 m seawater of ^12^C-AVFA incubation, **(B)** bottom 16 m seawater of ^12^C-AVFA incubation, **(C)** surface 2 m seawater of ^13^C-AVFA incubation, **(D)** bottom 16 m seawater of ^13^C-AVFA incubation samples, and amino acid concentrations with time in the **(E)** surface 2 m seawater of no-AVFA control and **(F)** bottom 16 m seawater of no-AVFA control samples. Data points were presented as average ± absolute error of duplicate samples except control samples.

AV, FA, VF, and VFA produced during hydrolysis of AVFA (Liu et al., 2013) remained at low levels <0.012 μM throughout the incubation, but more amino acids and peptide fragments were produced in the surface than in the bottom incubation (**Figure 2**). Concentrations of amino acid F were significantly higher in the surface than in the bottom seawater (*t*-test, *p* = 0.04). Concentrations of free amino acids (A, V, and F) were 2–30 times greater than those of peptide fragments. As individual amino acids have been shown in similar incubations to be taken up at different rates (Liu et al., 2013), particularly amino acid A was taken up much faster than V and F, production of amino acids might not follow the stoichiometry of added peptides. F was the dominant amino acid, reaching up to 0.17 μM in the surface at 24 h and 0.082–0.11 μM in the bottom at 8 h, and then decreased to the background level at the end of the incubation. V and A followed a similar pattern to F, but with smaller changes, indicating they were taken up faster than F, consistent with a previous study at this station (Liu et al., 2013). Compared to the AVFA treatment, concentrations of amino acids in the control without peptide amendment remained relatively low (<0.011 μM) and constant throughout the incubation.

Ammonium is a key metabolite of peptides (Liu et al., 2013). During the first 24 h in the surface seawater incubation, ammonium concentrations increased by 0.66–1.45 μM in the ^12^C- and ^13^C-AVFA samples (Supplementary Figures S1A–C). In contrast, ammonium concentrations in the bottom seawater changed little before AVFA was completely degraded (0–13 h). However, the difference of produced ammonium between surface and bottom seawater was not significant (*t*-test, *p* = 0.38). After 13 h, ammonium concentrations kept increasing to 2.6–2.9 μM in the surface seawater and remained constant at about 2.8 μM or increased by 1.5 μM to reach 4.1 μM in the bottom seawater. In the control without AVFA, ammonium concentrations increased by 0.97 μM in the surface seawater and decreased by 0.63 μM in the bottom seawater during the 48 h incubation.

P_i_ is an essential element for bacterial growth (Elser et al., 2000; Karl, 2014; Liu and Liu, 2016). However, P_i_ concentrations remained relatively constant throughout the 48-h incubation in both the peptide and control treatments (Supplementary Figures S1D–F). P_i_ concentrations in the bottom water (1.1–1.5 μM) were more than one order of magnitude higher than those in the surface water (0.02–0.09 μM).

### Bacterial Abundance and Community Structure

**FIGURE 3:**
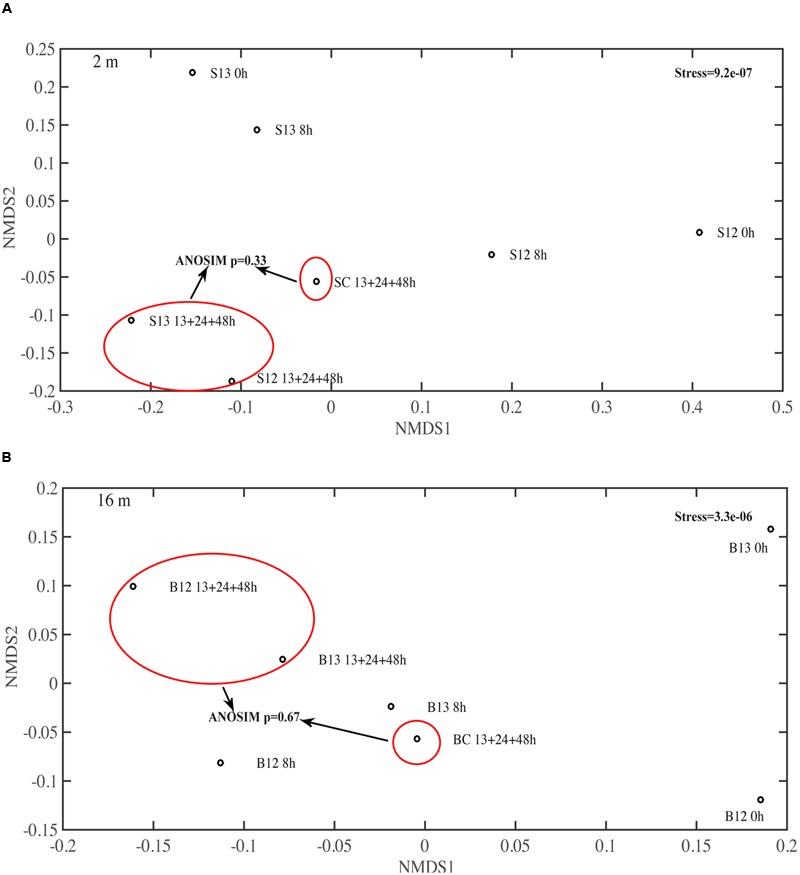
**Non-metric multidimensional scaling (NMDS) on the bacterial community structure at genera level in (A)** surface 2 m and **(B)** bottom 16 m time-point samples. 13, 24, and 48 h samples were pooled to be consistent with SIP data. S12, surface 2 m ^12^C-AVFA samples; S13, surface 2 m ^13^C-AVFA samples; SC, surface 2 m no-AVFA control samples; B12, bottom 16 m ^12^C-AVFA samples; B13, bottom 16 m ^13^C-AVFA samples; BC, bottom 16 m no-AVFA control samples. Stress was 9.2e-07 at 2 m and 3.3e-06 at 16 m, indicating excellent fitting of solution to recreate the dissimilarity (stress < 0.02). Bacterial composition formed separate clusters between unamended control and AVFA incubations at the later time points at both 2 and 16 m as shown in red circles, but ANOSIM showed this separation was not significant (*p* > 0.05).

In the surface ^12^C- and ^13^C-AVFA incubations, bacterial abundance increased by 31–57% within the initial 8–13 h, and then decreased afterward, while in the bottom, bacterial abundance increased by 44–45% during the initial 24 h and then decreased afterward (**Figures 1D,E**). Bacterial abundances in the control either decreased over time in the surface seawater or remained nearly constant in the bottom seawater (**Figure 1F**). Ambient surface water bacterial communities were dominated by *Synechococcus* (15–49%), whereas bottom samples were more evenly populated by *Rhodobacteraceae* (11–13%), *Acidimicrobiaceae* OCS155 marine group (3–8%), *Saprospiraceae* (5–7%), *Planctomycetaceae* (2–7%), SAR11 clade Surface 1 (3–6%), and *Acidimicrobiales* TM214 (3–5%) (Supplementary Figures S2A,B). Different initial surface community structures between ^12^C-AVFA and ^13^C-AVFA samples might be related to the loss of one duplicate ^12^C-AVFA DNA sample or contamination of *Synechococcus* in one sample. Thus, initial surface community structure of the ^13^C-AVFA duplicate samples might be more reliable than that of the ^12^C-AVFA samples. Throughout the incubation, the relative abundance of *Rhodobacteraceae, Thalassococcus*, and *Ruegeria* increased by 6–14, 7–13, and 1–3%, respectively, in both surface and bottom seawater, while other bacterial genera developed differently in the surface and bottom seawater incubations. For instance, OTUs classified within the *Roseovarius* clade increased by 7% only in the surface seawater, whereas *Colwellia* increased by 3% only in the bottom seawater. The surface and bottom bacterial community structures were well-separated in the NMDS plot (Supplementary Figure S2C); ANOSIM showed significant difference between the surface and bottom bacterial community structures (*p* = 0.002), further suggesting that bacterial community structures developed differently between the two water layers. To further evaluate the bacterial community development in unamended controls vs. AVFA treatments, NMDS was also plotted separately on surface and bottom bacterial communities (**Figure 3**). At the later incubation time points, unamended treatments and AVFA treatments formed separate clusters especially at NMDS1 axis, but this separation was not significant as shown in ANOSIM analysis (*p* > 0.05), indicating peptide amendement did not significantly change bacterial community structures during the 48-h incubation as compared to controls.

### Identifying Bacteria that Incorporated Peptides through DNA-SIP

**FIGURE 4:**
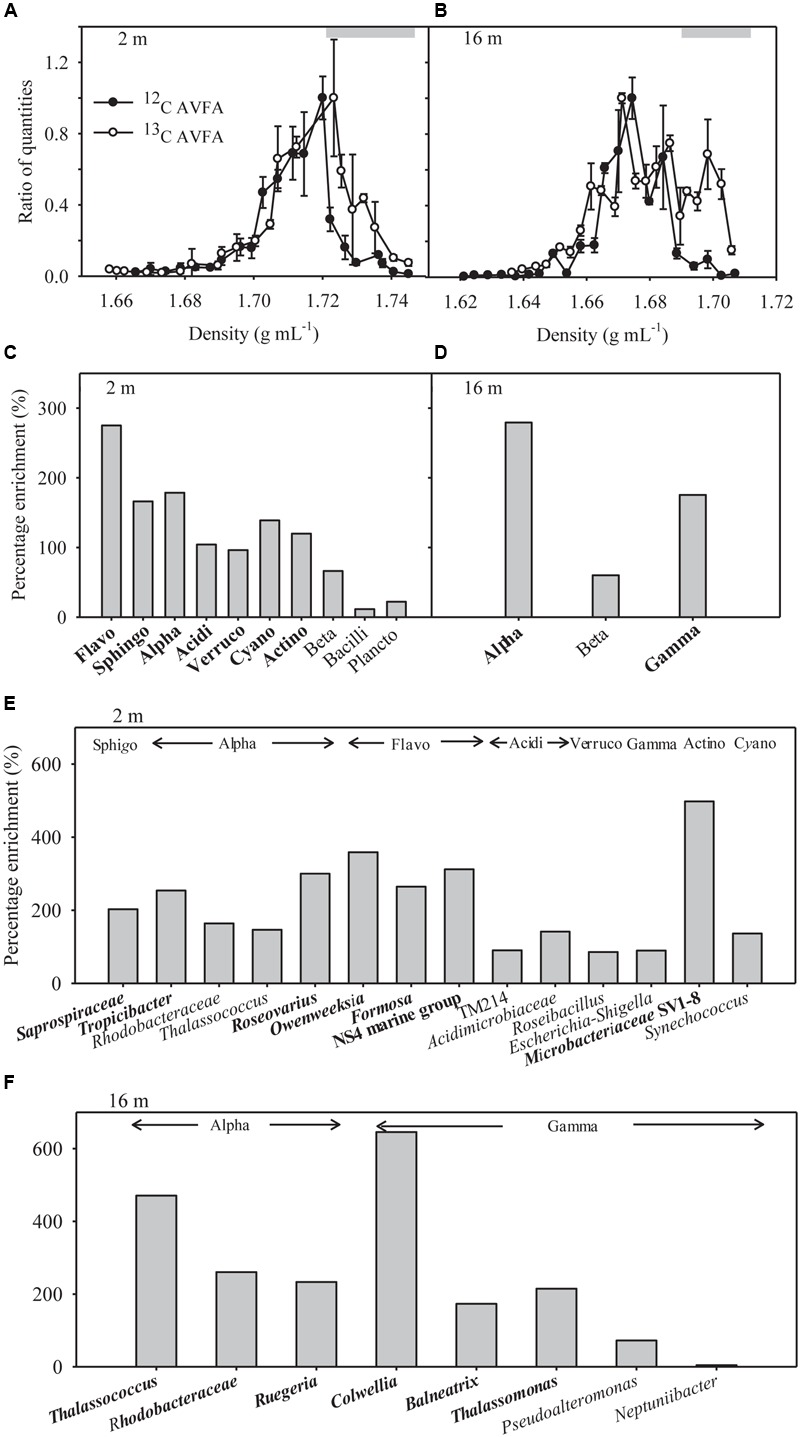
**(A,B)** Quantitative polymerase chain reaction (qPCR) analysis results shown as relative quantities vs. density of SIP gradient fractions for bacterial 16S rRNA gene copies in the surface 2 m and bottom 16 m samples. The ratio of quantities was calculated as 16S rRNA gene copy numbers in specific fraction normalized to the highest quantities of 16S rRNA gene copy numbers observed among all fractions in that sample, and 1 equals the highest value observed. Data points were presented as average ± standard deviation of three replicate qPCR measurements. Gray bars indicate heavy density ranges used for percentage enrichment calculations in **(C–F)**. **(C,D)** Percentage enrichment of major bacterial classes in the heavy density range in the ^13^C-AVFA SIP fractions compared to the ^12^C-AVFA SIP fractions. The 13, 24, and 48 h DNA samples were pooled together for SIP results. Bacterial class chosen were at least 0.1% abundance of the community. Flavo, Flavobacteria; Sphingo, Sphingobacteria; Alpha, Alphaproteobacteria; Acidi, Acidimicrobiia; Verruco, Verrucomicrobiae; Cyano, Cyanobacteria subsection I; Actino, Actinobacteria; Beta, Betaproteobacteria; Plancto, Planctomycetacia; Gamma, Gammaproteobacteria. Class with percentage enrichment >84% at 95% confidence interval was in bold. **(E,F)** Percentage enrichment of major bacterial genera within each class (listed above the bars) in the heavy density range of the ^13^C AVFA sample SIP fractions compared to the ^12^C AVFA sample SIP fractions in the surface 2 m and bottom 16 m seawater. Bacterial genera chosen were at least 0.1% abundance of the community. Bacterial class abbreviation was same as before. Genus with percentage enrichment >168% at 95% confidence interval was in bold.

Quantitative polymerase chain reaction of SIP fractions indicates that the density distribution of the 16S genes in bulk DNA of the ^13^C-AVFA samples shifted to heavier densities as compared to the ^12^C-AVFA samples in both the surface and bottom incubations (**Figures 4A,B**). The 16S PCR products from respective fractions were bar-coded and sequenced using Illumina Miseq to generate sequence libraries. A positive percentage enrichment is an indicator of the bacterial potential in incorporating ^13^C (Bell et al., 2011). Estimated error/noise of percentage enrichment was 46 and 65% at the class and genus level, respectively, as calculated from the 95% confidence interval. From this error/noise estimate, percentage enrichment above 84% at the class level and above 168% at the genus level was above the 95% confidence interval. ^13^C uptake, as suggested by SIP, was more evenly distributed among the bacterial classes in the surface seawater than in the bottom seawater (**Figures 4C,D**). Flavobacteria, Sphingobacteria, Alphaproteobacteria, Acidimicrobiia, Verrucomicrobiae, Cyanobacteria, and Actinobacteria showed highest enrichment ranging from 96 to 275% in the surface water, whereas Alphaproteobacteria and Gammaproteobacteria showed highest enrichment ranging from 175 to 279% in the bottom water.

Communities taking up ^13^C in the surface and bottom seawater also differed at the level of dominant genera (>0.1% of the total bacterial community) (**Figures 4E,F**). In the surface seawater, *Saprospiraceae, Tropicibacter, Roseovarius, Owenweeksia, Formosa, Flavobacteria* NS4 marine group, and *Microbacteriaceae* SV1-8 dominated the ^13^C uptake. In the bottom samples, major ^13^C enriched groups included *Thalassococcus, Rhodobacteraceae, Ruegeria, Colwellia, Balneatrix*, and *Thalassomonas*. The extent of taxonomic enrichment in the heavier fractions varied widely among different bacterial genera, ranging from 86 to 498% in the surface incubation and from 4 to 646% in the bottom incubation. Within the same class, the enrichment of *Roseovarius* and *Thalassococcus* was almost twice as high as that of other genera in Alphaproteobacteria, and the enrichment of *Colwellia* was more than three times higher than that of other genera in Gammaproteobacteria.

## Discussion

### Faster AVFA Decomposition in the Hypoxic than in the Normoxic Seawater

Peptide decomposition in the bottom incubation was twice as fast as that in the surface incubation (**Figures 1A,B**). Normalized to initial bacterial abundance, cell-specific rate of peptide decomposition in the bottom incubation (9.0 × 10^-9^–1.5 × 10^-8^ nM h^-1^) was 1.3–1.4 times as high as that in the surface incubation (6.4 × 10^-9^–1.2 × 10^-8^ nM h^-1^). Also, AVFA decomposition produced less hydrolyzed fragments, including amino acids and peptides, in the bottom than surface incubations (**Figure 2**), indicating direct uptake of AVFA or tightly coupled hydrolysis-uptake in the bottom water but extracellular hydrolysis in the surface water. In contrast to previous studies, which used relatively high concentrations of added peptides (5–10 μM) (Liu et al., 2013; Liu and Liu, 2016), the much lower concentrations of AVFA (0.25–0.47 μM) added here accounted for only 14–84% of ambient DCAA that consist of all hydrolyzable proteins and peptides in seawater (**Table 1**). As individual peptides may exist only at trace levels in ambient seawater, adding low concentration of peptides may simulate natural processes better. However, the low concentration amendments conducted here resulted in uptake patterns generally consistent with previous studies (Liu et al., 2013; Liu and Liu, 2016). Peptide decomposition all followed zero-order reaction with a linear decrease of concentration with time, indicating that the peptide decomposition in this study and previous studies was limited by the availability of enzymes produced by bacteria and the added peptide concentrations were probably all above a threshold of enzyme capacity in the ambient seawater. Faster peptide decomposition and less fragments produced in the bottom than in the surface incubations at both high and low added concentrations suggest that added peptides within this concentration range (0.25–10 μM) may trigger similar peptide decomposition mechanisms and bacterial response.

**FIGURE 5:**
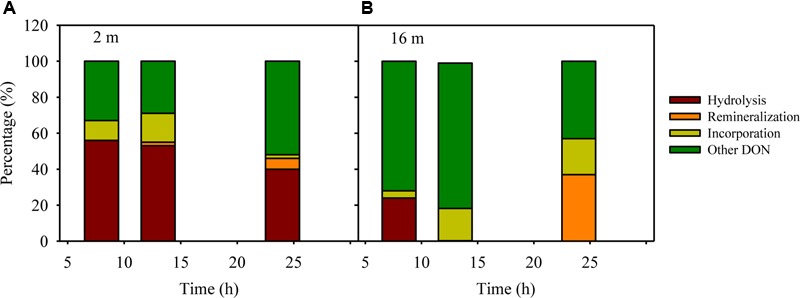
**An example of mass balance (including percentages of decreased peptide due to hydrolysis, remineralization to ammonium, incorporation into bacterial biomass and other unaccounted transformation to DON besides AVFA and amino acids) throughout the ^12^C-AVFA decomposition in the (A)** surface (2 m) and **(B)** bottom (16 m) seawaters.

The peptide decomposition mechanism can be interrogated through a mass balance of the fate of added nitrogen, which may include: (1) extracellular hydrolysis to produce peptide fragments and amino acids, (2) remineralization to ammonium, and (3) incorporation into bacterial biomass. The percentage of extracellular hydrolysis can be estimated using the amino acid F and peptide fragments containing F, as bacterial uptake of F is limited within 24 h (Liu et al., 2013). The degree of remineralization can be estimated via changes in ammonium concentrations in peptide treatments compared to controls assuming nitrification is negligible during the 24 h (Liu et al., 2013). To calculate the incorporation percentage to microbial biomass, we assume a carbon conversion value of 20 fg C per bacterial cell and a C/N ratio of 4 for bacteria (Lee and Fuhrman, 1987). Based on these parameters, extracellular hydrolysis (40–56%) dominated the decomposition of AVFA in the surface water, whereas biomass production (4–20%) dominated in the bottom water throughout the incubation, leaving a major fraction (29–81%) of the AVFA nitrogen uncounted for in both layers, possibly in other forms of DON (**Figure 5**), which might be semi-labile and refractory DON formed via microbial production or transformation processes (Jiao et al., 2010; Benner and Amon, 2015; Walker et al., 2016). For example, at 24 h, ca. 40% of decreased AVFA in the surface seawater was hydrolyzed to peptide fragments and amino acids, ca. 6% was converted to ammonium, 2–11% to bacterial biomass (8.1 × 10^4^–3.3 × 10^5^ cells per mL), and about 50% to other DON. In contrast, in the bottom seawater, less than 5% was hydrolyzed to peptide fragments and amino acids, hardly any ammonium was produced, and 18–28% was incorporated into bacterial biomass (7.7 × 10^5^–9.0 × 10^5^ cells per mL) at 13 h when AVFA disappeared, resulting in about 70–80% of AVFA nitrogen as other DON. This contrasting pattern suggests that the fast disappearance of AVFA in the bottom water incubation may relate to the higher percentage of peptide incorporation into bacterial biomass, i.e., bacterial growth. The efficiency of AVFA decomposition may depend on the fraction of nitrogen allocated to those fast-growing bacteria.

### Uptake of Peptide in the Normoxic vs. Hypoxic Seawater

In the surface seawater incubation, the incorporation of ^13^C was greatest for Flavobacteria, Sphingobacteria, Alphaproteobacteria, Cyanobacteria, Acidimicrobiia, Verrucomicrobiae, and Actinobacteria (**Figure 4C**), indicating that both oligotrophic (such as Cyanobacteria) and copiotrophic bacteria were involved in peptide decomposition in the surface seawater. Copiotrophic, perhaps *r*-selected, bacteria use labile organic matter in nutrient-enriched environments. This is in contrast to more *K*-selected oligotrophic species that maintain efficient metabolism by growing more slowly on complex refractory substrates (Fierer et al., 2007; Wang et al., 2015). At the genus level, *Saprospiraceae* (Sphingobacteria), *Tropicibacter* (Alphaproteobacteria), *Roseovarius* (Alphaproteobacteria), *Owenweeksia* (Flavobacteria), *Formosa* (Flavobacteria), Flavobacteria NS4 marine group (Flavobacteria), and *Microbacteriaceae* SV1-8 (Actinobacteria) took up the most ^13^C in the surface seawater (**Figure 4E**). Sphingobacteria showed a responsive role during peptone incubation in the seawater (Simon et al., 2012). The *Roseobacter* clade is often associated with plankton aggregates (Moran et al., 2007; Teeling et al., 2012; Yau et al., 2013), so *Tropicibacter* and *Roseovarius* belonging to the *Roseobacter* clade may be opportunistic in nutrient exploitation. Therefore, the observation that these populations can utilize the added peptide is expected. Flavobacteria are often effective in degrading high-molecular-weight DOM including proteins (Cottrell and Kirchman, 2000). Some Actinobacteria can produce a wide range of bioactive metabolites including extracellular peptidases that are sometimes involved in pathogenic processes (Ventura et al., 2007; Chen et al., 2011), suggesting their potential in peptide utilization. Consistent with our results, Orsi et al. (2016) also found that diverse bacterial taxa, such as Flavobacteria, Verrucomicrobia, Gammaproteobacteria, Alphaproteobacteria, Actinobacteria, and Planctomycetes, utilized added dissolved proteins in coastal California waters. The phylogenetic widespread of bacterial classes incorporating peptides in this study agrees with ecological theory and previous studies indicating that heterogeneity of the coastal oceans favors generalist bacteria in DOC utilization (Mou et al., 2008). Alternatively, this diverse bacterial pattern may result from the significant production of individual amino acids from extracellular hydrolysis (**Figures 2A,C**). Since uptake of amino acids is generally constitutive among marine bacterial taxa (Payne and Gilvarg, 1971; Poretsky et al., 2010) and uptake of amino acids is also part of peptide metabolizing process, bacterial groups possessing the ability to take up amino acids A, V, and F should be widespread, thus increasing the range of bacteria taxa showing positive percentage enrichment in the surface seawater.

In contrast to the surface seawater incubation, bacteria incorporating ^13^C in bottom waters were associated with fewer taxonomic groups, primarily belonging to the Alphaproteobacteria and Gammaproteobacteria (**Figure 4D**). The bacteria that metabolized the peptide differed between the surface normoxic and bottom hypoxic seawater. This depth-differential response was strikingly consistent with the study of Nelson and Carlson (2012), showing that while both oligotrophic and copiotrophic bacteria incorporated amended DOC sources such as Synechococcus exudate, Synechococcus lysate, and gluconic acid in the euphotic seawater, no oligotrophic bacteria showed evidence of incorporation of amended DOC sources in the mesopelagic seawater. This concomitant pattern in different seawater systems implies that different water parameters between depths are likely to be the driving force in bacterial response to DOM sources. The dominant percentage enrichment of Alphaproteobacteria and Gammaproteobacteria in the bottom seawater suggested they can outcompete other bacteria in incorporating AVFA, thus leading to the faster decomposition of the peptide in the bottom seawater. This result is consistent with previous studies in DOM utilization, which quantified this process either directly through tracing radioisotope incorporation by bacteria or indirectly via analyzing changes of bacterial community structure (Gihring et al., 2009; McCarren et al., 2010; Carney et al., 2015). For example, the percentage of Gammaproteobacteria consuming proteins was higher than their abundance percentage among all bacterial phylogenetic groups in estuarine and coastal environments, indicating they are efficient at metabolizing proteins (Cottrell and Kirchman, 2000). Alphaproteobacteria or Gammaproteobacteria can dominate the bacterial community during DOM incubation in certain marine environments, indicating they can outcompete other bacteria in using DOM substrates (Harvey et al., 2006).

At the genus level, the highest percentage enrichment in the bottom seawater occurred to the *Thalassococcus* (Alphaproteobacteria), *Rhodobacteraceae* (Alphaproteobacteria), *Ruegeria* (Alphaproteobacteria), *Colwellia* (Gammaproteo-bacteria), *Balneatrix* (Gammaproteobacteria), and *Thalassomonas* (Gammaproteobacteria) (**Figure 4F**). *Thalassococcus* has been shown to be capable of utilizing phthalate (Iwaki et al., 2012), but its ability to metabolize peptides, as suggested here, has not yet been explored. Previous studies have shown that *Rhodobacterales* are often one of the dominant groups in coastal seawaters, accounting for as high as 75% of the Alphaproteobacteria (Dong et al., 2014). Their abundance is thought to be related to DOC concentrations in nutrient-enriched habitats and they are frequently involved in taking up labile organic molecules, such as peptides and amino acids, as detected by metaproteomics (Dong et al., 2014; Fodelianakis et al., 2014). Our previous study also showed that populations of *Ruegeria, Thalassomonas, Pseudoalteromonas*, and *Neptuniibacter* grew rapidly when AVFA was amended to the same Sta. C6 bottom water (Liu et al., 2013). These genera contain many copiotrophs. Copiotrophs, such as *Ruegeria, Vibrio, Alteromonas*, and *Colwellia*, grow rapidly when substrates are available, but also maintain growth potential under starvation conditions. This is akin to a “feast or famine” strategy that allows adaptation to rapidly changing environments (Eilers et al., 2000; Christie-Oleza et al., 2012). Their high capability to assimilate peptide is thus consistent with their ecology strategy. The growth of *Pseudoalteromonadaceae* and *Colwellia* increased when peptone was incubated in the Southern Ocean seawater (Simon et al., 2012). Consistently, copiotrophic bacteria, such as *Vibrio, Roseobacter, Pseudoalteromonas, Photobacterium, Marinomonas, Marinobacter*, and *Alteromonas*, dominated the incorporation of DOC sources from *Synechococcus* exudate or lysate in seawater culture incubations (Nelson and Carlson, 2012). Particle-attached *Colwellia* and *Pseudoalteromonas* also showed high incorporation of proteins in marine microcosms (Mayali et al., 2015). DOC-related transporter genes, such as amino acids, oligopeptides, carbohydrates, carboxylic acids, polyamines, and lipids transporters, in coastal seawater were associated with *Rhodobacterales* (p*rimarily Roseobacter*), *Rickettsiales, Flavobacteriales*, and five orders of *Gammaproteobacteria*, including *Alteromonadales, Oceanospirallales, Pseudomonadales, Vibrionales*, and an uncharacterized taxon related to sulfur-oxidizing symbionts (Poretsky et al., 2010). Most of these bacteria also assimilated the peptide used in our study.

Changes in bacterial community structure that developed through incubations were not significantly different among the peptide treatment and control samples (**Figure 3**, ANOSIM *p* > 0.05, Supplementary Figure S2). These data suggest that peptide addition at relatively low concentrations had a minimal effect on the overall community structure. This also highlights that bacterial community structure cannot necessarily be used to infer roles of individual bacterial populations in incubation experiments. In contrast, the SIP technique directly links bacterial taxa with a metabolic function such as peptide decomposition. A direct comparison between the relative change in bacterial community structure and AVFA utilizing taxa, as determined via SIP, points to the interpretation that abundant bacterial taxa are not necessarily the most active ones (**Table 2**). For instance, *Saprospricaea, Escherichia–Shigella, Balneatrix*, and *Thalassomonas* accounted for <2% of communities and changed only <1% throughout the incubation while their abundance enrichment in heavy SIP fractions was 90–215%. *Microbacteriaceae* remained below 5% and their abundance did not increase with time during incubations, while frequencies increase by nearly 500% in heavy SIP fractions from surface seawater incubations. Similar observations have been reported elsewhere (Zemb et al., 2012), and indicate that some bacteria can be highly enriched in ^13^C, but they may represent only a small proportion of the overall community. These rare bacteria may have long generation time with 10s of hour or more (Brock, 1971). During our short 48 h incubation, certain bacteria might be at lag phase of growth, which changed little in the community structure. For example, if these rare bacteria only doubled once during 48 h, their increase from ca. <1% to ca. <2% would not contribute much the overall community structure. Alternatively, these rare bacteria might have utilized the assimilated peptides mostly for respiration instead of for biomass building, leading to the mismatch between abundance and SIP incorporation. These data showed the potential role of some rare and uncultivable bacteria in peptide utilization, which is often overlooked based on bacterial community structure analysis. This uncoupling between microbial abundance and activity is also consistent with other studies showing uncoupled pattern between rDNA and rRNA for some bacterial populations (Campbell and Kirchman, 2013; Caruso et al., 2013; Hunt et al., 2013), reflecting bacterial activity rates are not necessarily correlated to their abundance and these two parameters may be controlled by different factors.

**Table 2:** Comparison between the relative percentage change of bacteria genera (average percentage at 13+24+48 h relative to percentage at 0 h in quasi-replicates (*n* = 4) of ^12^C-AVFA and ^13^C-AVFA treatments) in the bacterial community structure and the percentage enrichment of bacteria genera showing positive enrichment in the SIP heavy fractions.

Depth	Bacteria genus	Percentage change in community structure (%)	Percentage enrichment in SIP (%)
2 m	*Saprospiraceae*	0.3	**203**
	*Tropicibacter*	1.7	**254**
	*Rhodobacteraceae*	12.6	164
	*Thalassococcus*	12.0	147
	*Roseovarius*	1.6	**300**
	*Owenweeksia*	-3.6	**359**
	*Formosa*	-0.4	**264**
	*Flavobacteria* NS4 marine group	-0.7	**312**
	*Acidimicrobiia* TM214	-0.9	91
	*Acidimicrobiaceae*	-1.8	142
	*Roseibacillus*	1.7	86
	*Escherichia–Shigella*	0.1	90
	*Microbacteriaceae* SV1-8	-0.7	**498**
	*Synechococcus*	-1.5	136
16 m	*Thlassococcus*	8.0	**471**
	*Rhodobacteraceae*	10.2	**261**
	*Ruegeria*	2.2	**233**
	*Colwellia*	3.2	**646**
	*Balneatrix*	0.2	**173**
	*Thalassomonas*	1.0	**215**
	*Pseudoalteromonas*	0.2	72
	*Neptuniibacter*	0.5	4

*Percentage enrichment above 168 at 95% confidence interval was in bold.*

### Factors Leading to the Development of Different Bacterial Communities

It is intriguing that bacterial communities that incorporated the added peptide differed in surface and bottom incubations. The two layers differed in chemical and biological parameters (**Table 1, Figures 1D–F, 3**, and Supplementary Figure S2), such as DO, DOC, and initial bacterial community structure, which probably contributed to the development of different bacterial communities, but the role of these factors seems to be limited (Liu et al., 2013; Liu and Liu, 2016). Other than these parameters, high levels of P_i_ (>0.4 μM) in the bottom seawater may stimulate the growth of fast-growing bacteria with high RNA content (Liu and Liu, 2016), such as Alphaproteobacteria and Gammaproteobacteria, consistent with the Growth Rate Hypothesis (Elser et al., 2000; Makino et al., 2003). The fast-growing bacteria may lead to faster peptide decomposition observed in the bottom than in the surface seawater. Assuming 0.2 pg dry mass bacterial cells and a P content of 1.3% (Sterner and Elser, 2002), the bacterial abundance increase observed here would have required 0.01–0.08 μM P_i_. These small values are close to the standard deviation (ca. 0.02 μM) of P_i_ measurement, which may explain why no obvious decrease of P_i_ was observed during our incubations (Supplementary Figures S1D,E). On the other hand, these results further suggest that the level of P_i_, rather than its absence, is the key factor limiting the development of fast-growing bacteria, supporting our previous hypothesis (Liu and Liu, 2016). The unique development of certain alphaproteobacterial and gammaproteobacterial genera may also explain the much lower production of AVFA fragments during the bottom water incubation compared to the surface water incubation (**Figures 2B,D**). Either these bacteria directly took up the peptide, or the hydrolysis and subsequent uptake of the fragments were tightly coupled (Fuhrman, 1987; Kuznetsova and Lee, 2002; Liu et al., 2013). These two processes cannot be differentiated with these data, but regardless, both pathways differ from that of the surface incubation, where hydrolysis and uptake seem uncoupled.

### Factors to be Considered for the DNA-SIP Approach

A successful DNA-SIP experiment depends on the amount of isotopically labeled substrate being assimilated and the length of the incubation time (Radajewski et al., 2003; Neufeld et al., 2007a). The substrate concentration must be high enough to ensure sufficient isotopic labeling of nucleic acids relative to unlabeled background substrates that are relatively abundant. However, if the substrate concentrations are too high, the incubation may deviate from the *in situ* situation. In our incubations, we added relatively low concentrations (0.25–0.47 μM) of AVFA to minimize disturbance to the natural substrates. qPCR results support the notion that sufficient isotope was incorporated into bacterial DNA. Successful uptake of peptide by bacteria is also indicated through the increased bacterial abundance in peptide treatments compared to control. Longer incubation time often results in greater isotope incorporation, but may also lead to cross-feeding, such as bacterial assimilation of labeled byproducts, intermediates or dead cells, produced from substrate metabolism (Neufeld et al., 2007a,c; Wang et al., 2015). To reduce cross-feeding, we applied relatively short incubation time (48 h) that was nonetheless sufficient to allow complete peptide loss.

A potential limitation of the DNA-SIP approach is that the buoyant density of DNA varies with G+C content. As G+C content may vary among different bacteria, this may result in a loss of power to identify bacteria that have incorporated the labeled substrate based on density shift (Buckley et al., 2007). However, it is more problematic for ^15^N than for ^13^C substrates given the greater buoyant density differential for nucleic acids labeled with ^13^C. The density shift in our results was >0.01 g mL^-1^, equating to ca. 28% of ^13^C incorporation, which is more than the minimum percentage (20%) that is typically required for separating ^13^C and unlabeled organisms (Uhlik et al., 2009). Note that the overall buoyant density differed somewhat between the surface and bottom DNA fractions (**Figures 4A,B**). It is unclear why this difference was observed, but may be related to the different bacterial community composition in the surface and bottom incubations, as %G+C contents of DNA vary among different bacterial taxa and higher %G+C leads to heavier density (Buckley et al., 2007; Holben, 2011). However, it is presumed that this density difference will not affect our ability to identify bacteria incorporating ^13^C, because the taxonomic percentage enrichment was derived relative to the corresponding ^12^C-AVFA incubations within the surface or bottom samples. We note that DNA from three incubation time points (13, 24, 48 h) was pooled. This approach results in ‘smearing’ of the signal by spreading the DNA of active bacterial taxa across the gradient density range to a greater degree than for a single time point. However, this smearing should not be problematic with respect to the objectives of this study, because major bacterial taxa in the bacterial community structure were similar at all these three time points and the chemistry data showed a continuous pattern among these three time points. AVFA were completely degraded during 24–48 h in the surface seawater and during 13–24 h in the bottom seawater. Bacterial cell replication and DNA synthesis may have time lag after peptide incorporation due to their 9–12 h generation time (Eilers et al., 2000). Bacterial abundance was still increasing after 13 h in the bottom seawater (**Figures 1D,E**), indicating bacteria were still utilizing peptides for their growth shortly after peptide was completely degraded. To be consistent between two depths and make sure enough ^13^C signal is obtained, pooling the last three time points seems an appropriate choice. As surface and bottom incubations were treated in the same way for SIP samples, the comparison between two waters still holds with pooled samples. While the exact degree of isotopic labeling or peptide incorporation may therefore not be attainable from our experiments, the high degree of enrichment observed for some bacteria (**Figures 4C–F**) supports the notion of active ^13^C incorporation.

## Conclusion

Work presented here builds on prior observations with respect to the inferred role of bacteria in peptide decomposition (Liu et al., 2013; Liu and Liu, 2016). Here, we directly linked specific bacterial taxa with peptide decomposition in surface and bottom waters in the hypoxic region of northern Gulf of Mexico. Major conclusions and implications from this study are as follows:

(1) Bacterial groups metabolizing peptide appear to differ between the surface normoxic and bottom hypoxic seawater. A more diverse group of bacteria including both oligotrophs and copiotrophs might be involved in peptide decomposition in the surface normoxic seawater, while peptide substrates appear to favor several copiotrophic marine bacterial lineages in the bottom hypoxic seawater. With a combination of detailed chemical and biological data in an isolation-independent way, this study expands our understanding of linkage between peptide decomposition and bacterial communities, especially with low concentrations of peptide amendment, and sheds new light on microbial behavior as single cells, populations and communities in microbial ecology.
(2) Peptide decomposition efficiency, pathway and fate differed between the surface normoxic and bottom hypoxic seawater, which might be related to the energy and element allocation to different bacterial taxa under distinct marine environments. By combining these chemical analyses with SIP results, the role of bacteria in contributing to this difference can thus be inferred. This serves as the first step to explore marine C and N cycle efficiency and mechanisms in various marine environments.
(3) This study further implies that some bacteria taxa can rapidly metabolize peptide in the context of high P_i_ concentration in the hypoxic seawater. It provides insights into the interactions among bacteria, labile DOM, nutrient, and DO in seawater, which stimulates more ecological hypotheses about diverse microbial groups and their functions in marine environments. As hypoxia may be intensified in the future scenario, investigating bacterial decomposition of labile DOM under different nutrient conditions is necessary to pinpoint the factors controlling hypoxia formation.

## Author Contributions

All authors listed have made substantial, direct and intellectual contributions to the work and approved its final version for publication.

## Conflict of Interest Statement

The authors declare that the research was conducted in the absence of any commercial or financial relationships that could be construed as a potential conflict of interest.

## Footnotes

1 http://www.arb-silva.de
