# Supplementary material for: Different Bacterial Communities Involved in Peptide Decomposition between Normoxic and Hypoxic Coastal Waters
Source: Front Microbiol. 2017 Mar 7;8:353. doi: 10.3389/fmicb.2017.00353 (PMC5339267; doi:10.3389/fmicb.2017.00353)
Supplement: Supplementary file 1 [file Image_1.pdf]

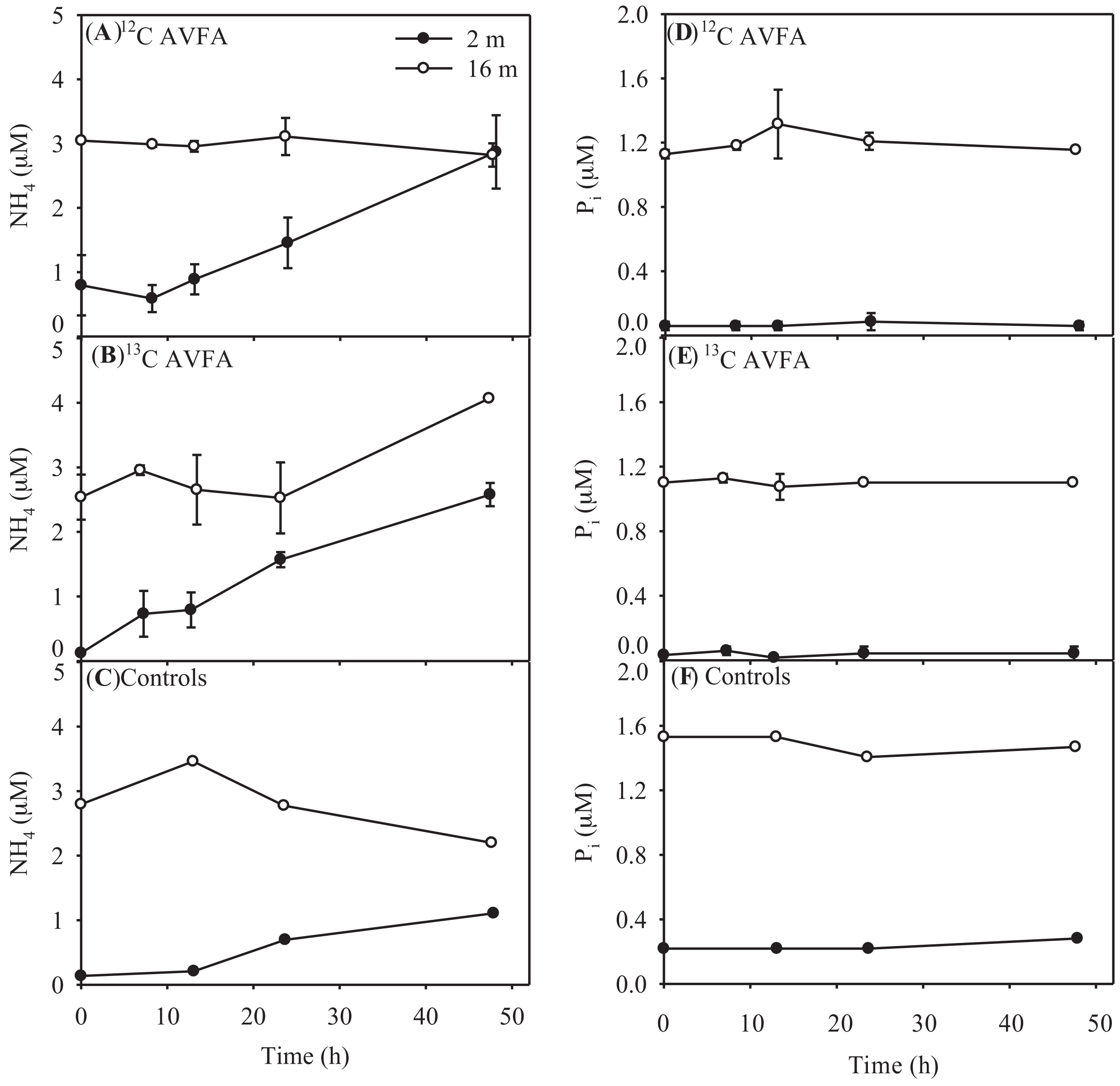

Figure S1. Concentrations of ammonium (a-c) and orthophosphate (Pi) (d-f) with incubation time in the surface 2 m and bottom 16 m seawater of ^{12}C-AVFA, ^{13}C-AVFA and no-AVFA control samples. Data points were presented as average ± absolute error of duplicate samples except control samples.
