# Supplementary material for: Different Bacterial Communities Involved in Peptide Decomposition between Normoxic and Hypoxic Coastal Waters
Source: Front Microbiol. 2017 Mar 7;8:353. doi: 10.3389/fmicb.2017.00353 (PMC5339267; doi:10.3389/fmicb.2017.00353)
Supplement: Supplementary file 2 [file Image_2.pdf]

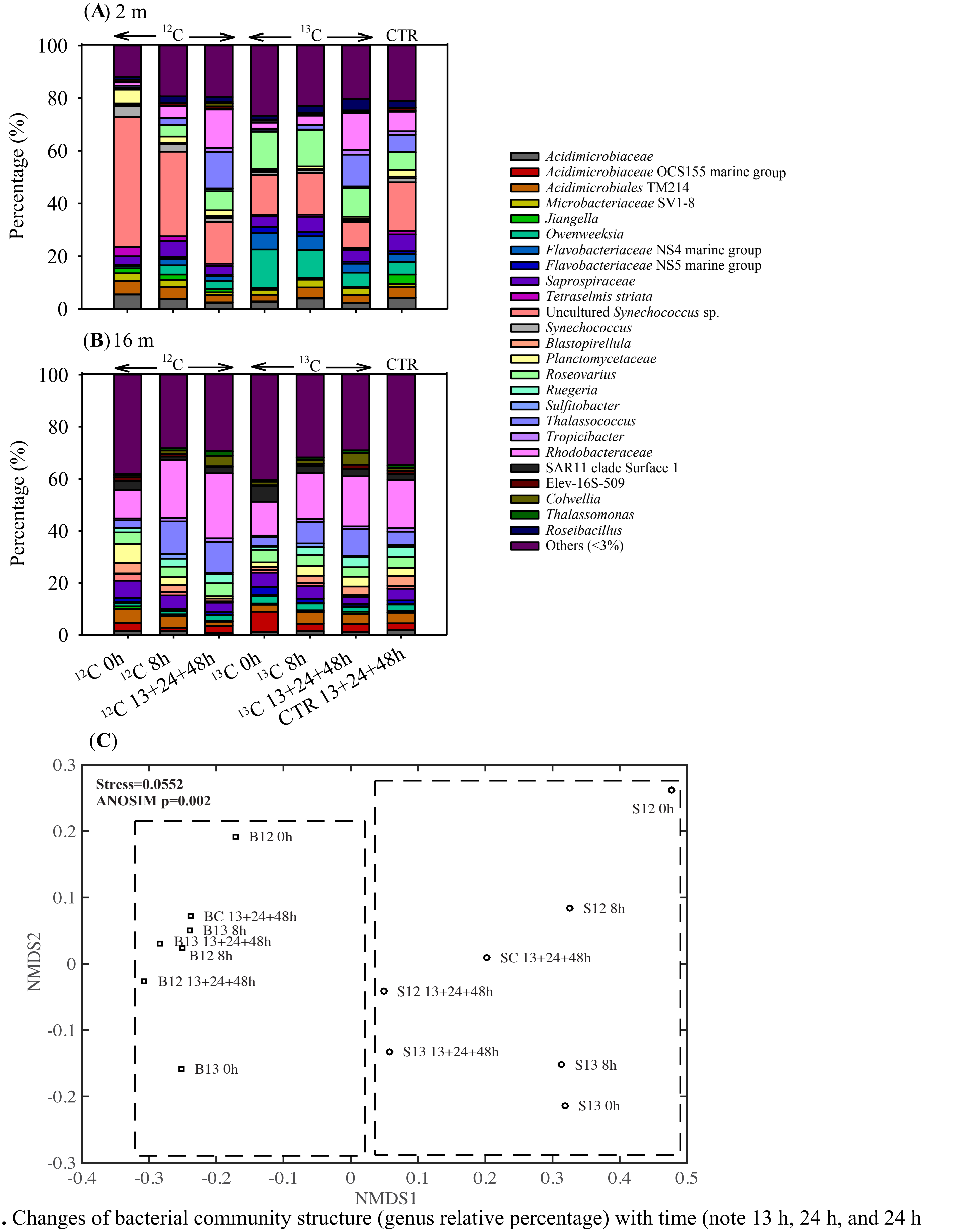

Figure S2. Changes of bacterial community structure (genus relative percentage) with time (note 13 h, 24 h, and 24 h samples were pooled to be consistent with SIP data) during ^{12}C-AVFA, ^{13}C-AVFA and no-AVFA control (CTR) incubation in the (A) surface 2 m and (B) bottom 16 m seawater; percentages were average of duplicate samples except control, 2 m 0 h and 16 m 0 h samples; (C) non-metric multidimensional scaling (NMDS) on the bacterial compositions at genera level in all the above samples; S12, surface 2 m ^{12}C-AVFA samples; S13, surface 2 m ^{13}C-AVFA samples; SC, surface 2 m no-AVFA control samples; B12, bottom 16 m ^{12}C-AVFA samples; B13, bottom 16 m ^{13}C-AVFA samples; BC, bottom 16 m no-AVFA control samples.
